# Non-bacterial thrombotic endocarditis in the right atrium caused by pectus excavatum

**DOI:** 10.1186/s40792-016-0236-4

**Published:** 2016-09-28

**Authors:** Ai Sugimoto, Shuichi Shiraishi, Maya Watanabe, Jiyong Moon, Riuko Ohashi, Masashi Takahashi, Masanori Tsuchida

**Affiliations:** 1 Division of Thoracic and Cardiovascular Surgery, Niigata University Graduate School of Medical and Dental Sciences, 1-757, Asahimachi-dori, Niigata City, 951-8510 Japan; 2 Division of Pathology, Niigata University Graduate School of Medical and Dental Sciences, Niigata, Japan

**Keywords:** Pectus excavatum, Cardiac thrombi, Non-bacterial thrombotic endocarditis

## Abstract

**Background:**

Non-bacterial thrombotic endocarditis (NBTE) is an uncommon pathological situation, which involves the presence of bland, fibrin-platelet thrombi. It usually occurs at the endocardium of cardiac valves, in association with endothelial injury and a hypercoagulative state. However, NBTE on the endocardium at the right atrial free wall in a patient without any apparent hypercoagulative background is rarely reported.

**Case presentation:**

A girl aged 4 years with severe pectus excavatum was referred to our hospital for treatment of a recurrent right atrial tumor. The tumor was removed concomitant with pectus excavatum repair. The tumor was revealed as recurrent thrombus. Pathological findings showed that NBTE caused by an operative scar on the endocardium of the right atrium and sustained rheological stress in the right atrium due to compression from pectus excavatum lead to recurrent thrombus formation. Three years after the discontinuation of anticoagulation therapy, no sign of thrombus formation was found.

**Conclusions:**

To our knowledge, this is the first report of NBTE related to an interaction between sustained rheological stress from cardiac compression and endocardial injury. In such patients, we recommend concomitant chest wall repair when the operative scar is present at the site of the rheological force.

## Background

Non-bacterial thrombotic endocarditis (NBTE) is an uncommon pathological situation, which involves the presence of bland, fibrin-platelet thrombi. NBTE usually occurs at the endocardium of cardiac valves, in association with endothelial injury and a hypercoagulative state. However, NBTE on the other location, or NBTE in patients without any apparent hypercoagulative background is rarely reported.

We report a case of NBTE, in a 4-year-old girl, caused by a combination of endothelial injury from a previous surgery and continuous endothelial damage and repair resulting from rheological force due to right atrial compression by severe pectus excavatum.

## Case presentation

**Fig. 1:**
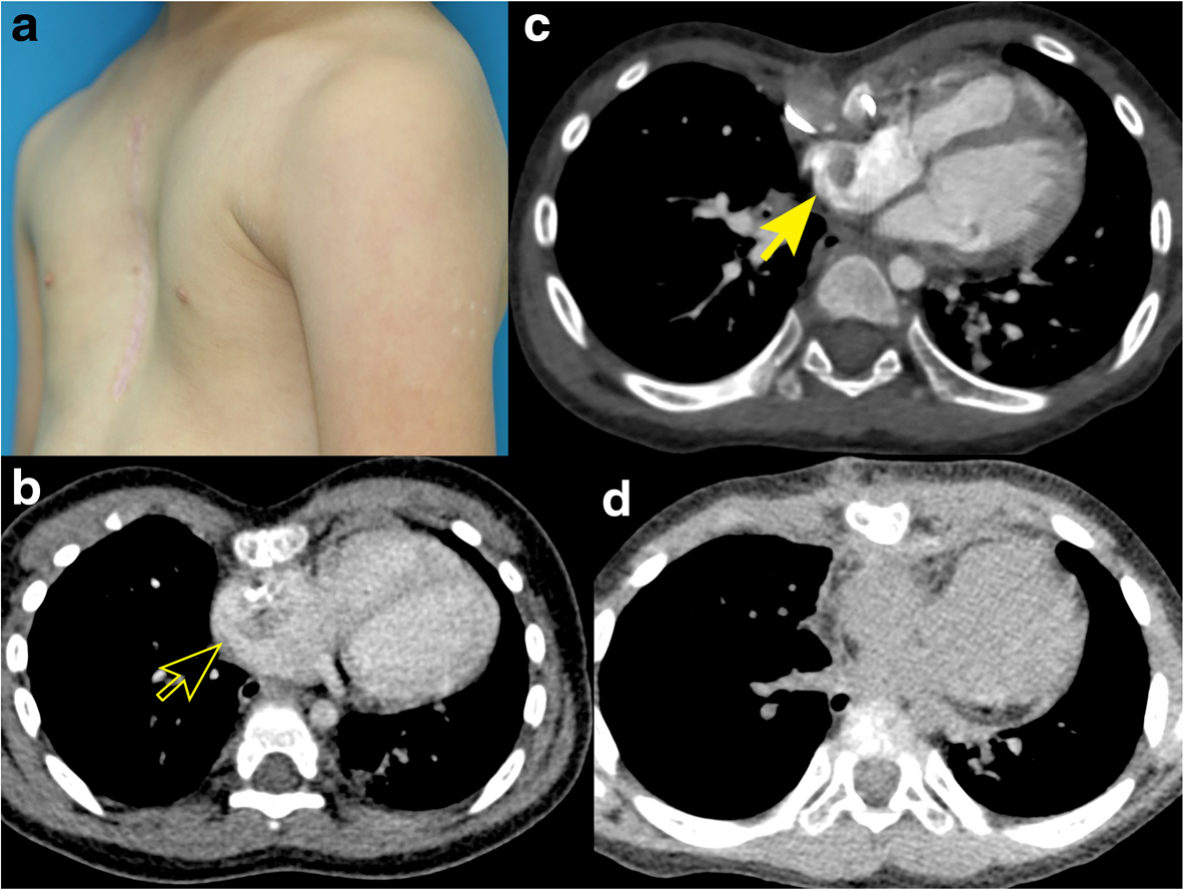
**a** Appearance of the patient’s chest at admission for the second removal for the recurrent right atrial thrombus (at the age 4.8 years): The chest is significantly excavated. **b** CT image at the time of the primary referral for the right atrial thrombus (at the age of 4 years): The tumor appears as a contrast defect in the right atrium (*black arrow* which was surrounded with *yellow*). The chest is significantly excavated. **c** CT image at the time of the second removal for the recurrent right atrial thrombus (at the age 4.8 years): the right atrium is externally compressed by the excavated chest. The tumor appears as a contrast defect in the right atrium (*yellow arrow*). **d** Postoperative CT image after the second right atrial thrombus removal concomitant with the pectus excavatum repair: the chest was well-repaired and no contrast defect was found in the right atrium

A girl with atrial septal defect (ASD), trisomy 21, and mild pectus excavatum underwent ASD patch closure at the age of 1 year. At the age of 4 years, she presented with a mass in the right atrium (RA) attached to the suture line of previous RA-tomy and severe pectus excavatum (Fig. 1b). The mass was removed promptly, and pathological studies identified it as a thrombus. Anticoagulation therapy with warfarin was started and was continued for 3 months. Three months after warfarin cessation, tumor recurrence was noted; therefore, she was referred to our department at the age of 4.8 years.

At admission, she was afebrile and physical examination showed normal findings. Her chest was severely excavated (Fig. 1a). No coagulation disorder was suspected, and laboratory findings did not suggest infection. Echocardiography revealed a pedunculated mobile tumor attached to the RA free wall. Computed tomography (CT) showed external compression of the RA (Fig. 1c). We speculated that the tumor was a recurrent thrombus. We planned to repair the pectus excavatum and remove the tumor simultaneously.

**Fig. 2:**
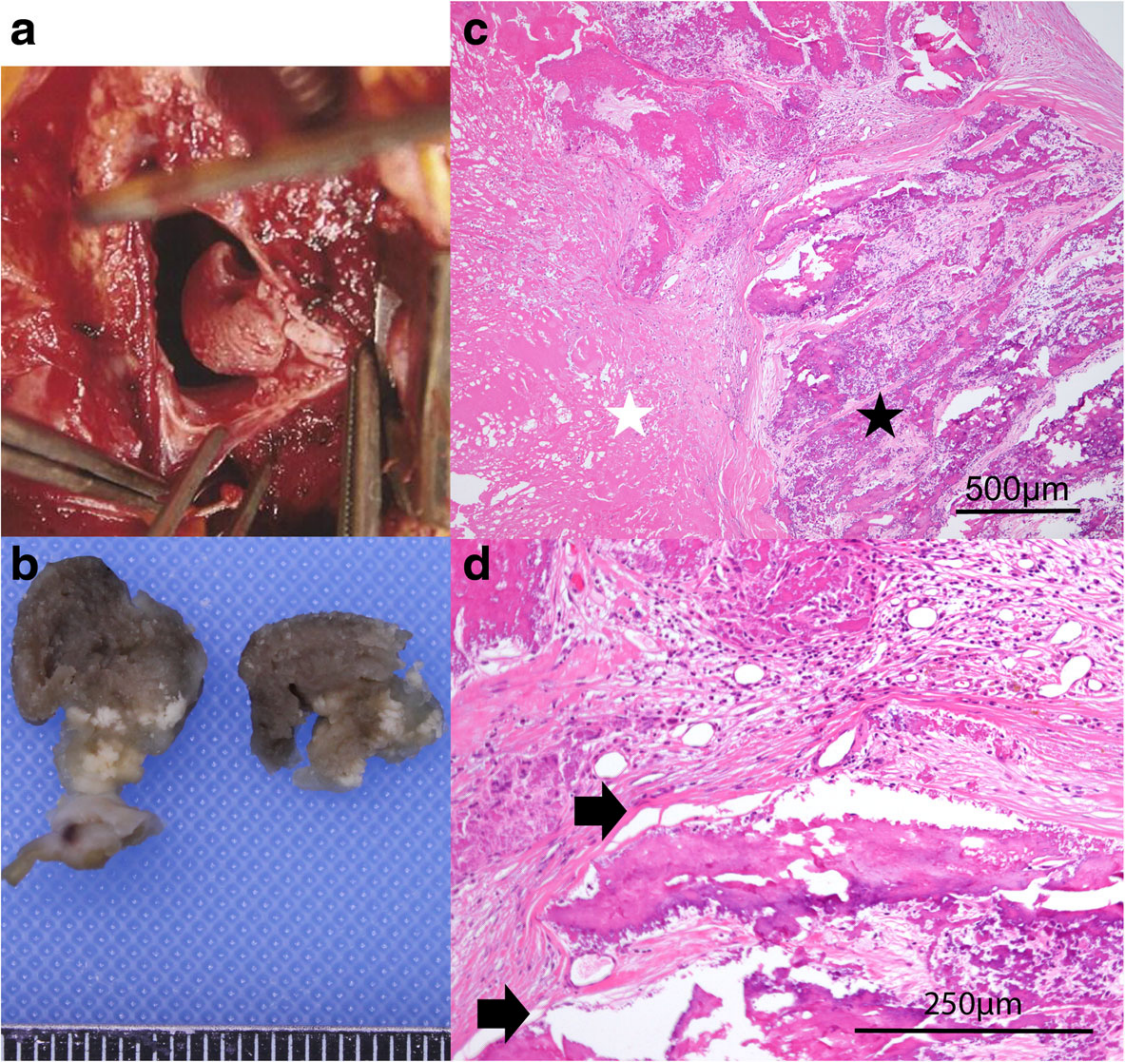
Intraoperative macro- and microscopic finding of the thrombus. **a** Intraoperative appearance of the right atrium tumor: A tumor is attached to the suture line of the previous right atriotomy. **b** Macroscopic finding of the tumor: the tumor was about 10 mm large in diameter. **c** Microscopic findings (hematoxylin and eosin stain, ×40): cicatricial fibrotic thickening and calcification of the endocardium (*black star*) and a platelet-fibrin thrombus (*white star*) are seen. **d** Microscopic findings (hematoxylin and eosin stain, ×100): granulation tissue formation with capillary vessels and calcification is seen between the platelet-fibrin thrombus and calcified and fibrotic thickened endocardium. Tissue damage is seen (*black arrows*), suggesting a persistent external force

During the operation, we noted that the tumor was attached to the suture line of the previous RA-tomy (Fig. 2a, b). The pectus excavatum was repaired using the modified Ravitch procedure. The total operation time was 6.5 h. Pathological studies identified the tumor as a thrombus, and anticoagulation therapy was restarted. Postoperative CT showed that her chest was repaired and the compression of the RA had been resolved (Fig. 1d). She was discharged on the 18th postoperative day. Further pathological studies confirmed NBTE. There was granulation tissue formation with capillary vessels and calcification between the platelet-fibrin thrombus and the calcified and fibrotic thickened endocardium. Additionally, there was tissue damage suggesting a persistent external force (Fig. 2c, d). Anticoagulation therapy was continued for 6 months postoperatively. The patient showed no sign of a recurrent RA thrombus 3 years after discontinuation of anticoagulation therapy.

### Discussion

To our knowledge, this is the first report of NBTE related to an interaction between sustained rheological stress from cardiac compression and endocardial injury.

NBTE is an uncommon pathological situation, which involves the presence of bland, fibrin-platelet thrombi, usually on valve tissue. It has been reported in approximately 0.3–9.3 % of autopsy patients [1] and has been reported to possibly cause major embolization from a cardiac valve thrombus, leading to significant morbidity and mortality [2].

The major factors associated with NBTE are endothelial injury and a hypercoagulative state [3]. In the present case, the tumor was attached to the suture line of previous RA-tomy; thus, endothelial injury might be related to NBTE. In addition, pathological study confirmed tissue damage suggesting a persistent external force. We assume continuous rheological force due to external RA compression by pectus excavatum at the site of the previous RA-tomy might have caused continuous endothelial damage at that location. Continuous endothelial injury and the subsequent repairing mechanism might have induced a hypercoagulative state, and fibrin-platelet deposition was believed to have occurred spontaneously.

The treatment for NBTE involves resolving the underlying process. In situations in which the underlying condition cannot be controlled rapidly, anticoagulation therapy with heparin is recommended [1]. In our patient, we achieved a good outcome by resolving the hemodynamic circumstances with pectus excavatum repair and adjunctive anticoagulation therapy for 6 months in the early postoperative period.

## Conclusions

We reported a rare case of NBTE related to an interaction between sustained rheological stress from cardiac compression and endocardial injury. In such patients, we recommend concomitant chest wall repair when the operative scar is present at the site of the rheological force.

## Abbreviations

ASD: Atrial septal defect
CT: Computed tomography
NBTE: Non-bacterial thrombotic endocarditis
RA: Right atrium
